# Refusal to enrol in Ghana’s National Health Insurance Scheme: is affordability the problem?

**DOI:** 10.1186/s12939-014-0130-2

**Published:** 2015-01-17

**Authors:** Anthony Kusi, Ulrika Enemark, Kristian S Hansen, Felix A Asante

**Affiliations:** Section for Health Promotion and Health Services Research, Department of Public Health, Faculty of Health, Aarhus University, Vennelyst Boulevard 6, 8000 Arhus C, Denmark; Department of Global Health and Development, Faculty of Public Health and Policy, London School of Hygiene and Tropical Medicine, 15-17 Tavistock Place, WC1H 9SH London, UK; Institute of Statistical, Social and Economic Research (ISSER), University of Ghana, P.O. Box LG 74, Legon, Accra, Ghana

**Keywords:** Voluntary health insurance, Universal coverage, Enrolment, Premium, Affordability, Ghana

## Abstract

**Background:**

Access to health insurance is expected to have positive effect in improving access to healthcare and offer financial risk protection to households. Ghana began the implementation of a National Health Insurance Scheme (NHIS) in 2004 as a way to ensure equitable access to basic healthcare for all residents. After a decade of its implementation, national coverage is just about 34% of the national population. Affordability of the NHIS contribution is often cited by households as a major barrier to enrolment in the NHIS without any rigorous analysis of this claim. In light of the global interest in achieving universal health insurance coverage, this study seeks to examine the extent to which affordability of the NHIS contribution is a barrier to full insurance for households and a burden on their resources.

**Methods:**

The study uses data from a cross-sectional household survey involving 2,430 households from three districts in Ghana conducted between January-April, 2011. Affordability of the NHIS contribution is analysed using the household budget-based approach based on the normative definition of affordability. The burden of the NHIS contributions to households is assessed by relating the expected annual NHIS contribution to household non-food expenditure and total consumption expenditure. Households which cannot afford full insurance were identified.

**Results:**

Results show that 66% of uninsured households and 70% of partially insured households could afford full insurance for their members. Enroling all household members in the NHIS would account for 5.9% of household non-food expenditure or 2.0% of total expenditure but higher for households in the first (11.4%) and second (7.0%) socio-economic quintiles. All the households (29%) identified as unable to afford full insurance were in the two lower socio-economic quintiles and had large household sizes. Non-financial factors relating to attributes of the insurer and health system problems also affect enrolment in the NHIS.

**Conclusion:**

Affordability of full insurance would be a burden on households with low socio-economic status and large household size. Innovative measures are needed to encourage abled households to enrol. Policy should aim at abolishing the registration fee for children, pricing insurance according to socio-economic status of households and addressing the inimical non-financial factors to increase NHIS coverage.

## Introduction

Access to health insurance is known to have positive effects in improving access to healthcare services and serves as a mechanism for avoiding catastrophic healthcare expenditures which often plunge low resourced households into poverty [1-3]. It is estimated that globally, about 150 million people face catastrophic healthcare costs annually because of direct payments for healthcare while about 100 million are driven into poverty [1]. One major global response to address this problem has been the implementation of prepayment schemes especially in low-and-middle income countries (LMICs) where majority of the population without health insurance lives [1,4,5].

Since the mid 1990s, several LMICs have been implementing various types of health insurance programmes as a response to the global effort to move away from the reliance on out-of-pocket payment for health towards risk-pooling and risk-sharing arrangements [1-3,6,7]. As was the trend in several African countries in the 1990s, Ghana began to witness the establishment of several community health insurance (CHI) schemes as part of what has been described as the ‘African CHI movement’ due to the rapidity of their growth on the continent [8,9].

In 2004, Ghana began the implementation of a National Health Insurance Scheme (NHIS) following the passage of the National Health Insurance Act (Act 650) in 2003. The NHIS has an overall goal of ensuring equitable access to quality basic healthcare for all residents, without having to make out-of-pocket payments at the point of service. Despite the huge potential benefits associated with access to health insurance, enrolment into the NHIS has not been as high as expected after a decade of implementation. Only 34% of Ghana’s population of 24.6 million are active members (i.e. valid card holding members) [10]. This figure is considered low given the fact that the NHIS premium and registration fee are relatively low and there is a comprehensive premium exemption package for a large section of the population. The low enrolment in the NHIS presents a major challenge towards achieving the goal of universal coverage [11].

Low enrolment in voluntary health insurance schemes in LMICs is common and has been attributed to several factors including the lack of adequate consumer information, lack of understanding of the insurance concept and the benefit package among the target population, lack of trust in insurers, perceived poor quality of available healthcare services, poverty, unaffordable premiums, unfavourable timing of the premium payment, institutional rigidities, large informal sector and low levels of education, among others [4,6,12-18]. While many of these factors have been observed in Ghana, affordability of the premium and registration fee (i.e. NHIS contributions) continues to be reported by non-members of the NHIS as the most important barrier to enrolment and retention in the NHIS [19-25].

Empirical studies in the United States of America (USA) have suggested that health insurance premiums could be affordable to most of the uninsured including some low income families [26-28]. What constitutes affordable premium however lacks a clear economic definition, though attempts have been made to estimate it in the developed world [27-29]. In the few studies in Africa which have looked at the premium as a barrier to enrolment or retention in health insurance schemes, attempts have not been made to estimate what constitutes affordable insurance contributions [13,14,30,31], though few have analysed household capacity to pay the insurance premium [32,33]. These studies have reported mixed results on whether affordability is the main barrier to enrolment. For instance, in a review of the impact of mutual health organisations in West Africa, Chankova et al. [34] report that premium payments can be unaffordable to many households even when small and can therefore become a major barrier to enrolment. Diop [32] however observed that the majority of households surveyed in the Thies region of Senegal had the ability to pay insurance contributions as the incidence of insurance contribution was about 1.2% of total household expenditures and about 5% of non-food expenditures. Findings from another study in Burkina Faso suggested that the low demand for community-based insurance may be due to institutional rigidities rather than to poverty per se [13].

Previous empirical studies on equity in the NHIS either at the individual or household levels have reported a strong association between high socio-economic status and NHIS membership suggesting that the ‘poor’ are excluded from the NHIS because they cannot afford membership [19,20,22,24,25]. However, in a recent anthropological study in the Central and Eastern regions of Ghana, Kotoh [21] observes that “enrolment does not neatly correlate with economic status” and that the “no money to pay premium response” often cited by majority of the uninsured is a “convenient excuse to rationalise non-enrolment and non-renewal of membership”. In another study on equity in the two main cities of Ghana, Amporfu [35] also reports that ‘the premium is likely to impose catastrophic expenditure on a small minority of the poor’.

The World Health Organisation (WHO) has recently observed that the current global coverage of financial risk protection falls far short of universal coverage [1,36]. In light of the global interest in achieving universal health insurance coverage especially in developing countries, Ghana needs to do more to achieve that goal by ensuring that households enrol all their members in the NHIS. This however raises a concern about affordability in the face of the numerous complaints about the NHIS contributions by households, though what constitutes affordable contributions to households have not been studied. This study examines the affordability of the NHIS contributions to households by assessing the reasons for not enrolling and by estimating the expected annual NHIS contributions for full insurance across uninsured, partially insured and fully insured households. This is aimed at understanding whether households in Ghana are ‘too poor’ to afford enrolment in the NHIS and the extent to which the NHIS contribution would be a burden on their resources. We also aimed at identifying households that cannot afford to have full insurance for their members.

The rest of the paper continues with a brief overview of the NHIS in section two, while section three deals with the methods; study design, data collection and statistical analyses. Results are presented in section four, while section five presents the discussion and conclusion.

### An overview of Ghana’s NHIS

The National Health Insurance Act (Act 650) of 2003 permits the establishment and operation of three types of health insurance schemes in Ghana. They include the district mutual health insurance schemes, private commercial health insurance schemes and private mutual health insurance schemes. The private commercial schemes operate as limited liability companies, while the private mutual schemes are organised by individual groups of persons for their own benefit. Both types do not receive any financial support from the government. By Act 650, districts are to establish their own district mutual health insurance schemes (DMHIS). The DMHIS are autonomous from each other but all operate under the National Health Insurance Authority (NHIA). The new NHI Act of 2012 (Act 852) merges all the DMHIS to form a nationwide National Health Insurance Scheme (NHIS) which every resident of Ghana shall belong to [37]. The NHIA provides subsidy from the National Health Insurance Fund (NHIF) to the DMHIS for their operations. The state sponsored DMHIS are the dominant health insurance schemes across the country and operational in all the districts of Ghana. A member of the NHIS may also belong to a private health insurance scheme as stipulated by Act 852. This study focuses on the NHIS comprising of the DMHIS because that is public and of national interest.

The NHIA has five main sources of funds for its operations. The NHI Levy which is a 2.5% value added tax (VAT) on selected goods and services which accounts for about 60% of the total revenue making it the major contributor to the NHIF. The other sources of revenue to the fund include a mandatory 2.5% deduction from formal sector workers’ social security contribution managed by the Social Security and National Insurance Trust (SSNIT), sector budgetary support allocated by the Parliament of Ghana, income accruing to the NHIF from investments made by the NHI Council and grants, fees, donations, gifts and voluntary contributions made to the Fund [37]. The premiums paid by members is another source of funds but this accounts for less than 5% of the total inflows to the NHIS [38].

Membership in the NHIS is at the individual level and supposed to be mandatory by law for all residents of the country. According to Act 852, all employers are also obliged to ensure that all their employees are registered under the NHIS. Individual adults aged 18–69 years in the informal sector pay annual premiums (i.e. direct premium-paying adults) as determined by the DMHIS and approved by the NHIA. The premium ranges between 7.2 Ghana Cedis (GhȻ) (US$4.8) to GhȻ48.0 (US$32) depending on the socio-economic status (SES) of the individual. However due to the difficulty in determining SES of people in the informal sector, the premium is in practice set at a flat rate and varies from district to district. Formal sector workers whose premiums are deducted from their social security contributions (i.e. SSNIT contributors) are exempted from direct premium payments to the scheme to become members. They are however not automatic members of the NHIS unless they decide to enrol with a DMHIS of their choice by paying a registration fee. In 2011, SSNIT contributors constituted only 4.3% of the total active membership of the NHIS [38].

The NHI Act 852 exempts children under 18 years from paying the premium if at least one parent or the guardian is a valid card holder of the NHIS (this clause in the LI 1809 has been scrapped by the new Act 852 of 2012 though the legislative instrument for its implementation is not yet out). The registration of children under five years has however been decoupled from that of their parents since 2010 and therefore they can be registered even if their parents are not registered. The elderly (≥70 years), SSNIT pensioners, core poor indigents identified by communities and pregnant women (for ante-natal, delivery and post-natal healthcare services) are also exempted from paying premiums to become members of the scheme. Apart from the indigents and pregnant women, all exempt populations are required to pay a registration fee of GhȻ4.0 (US$2.7) but the fee could vary depending on the district scheme.

The NHIS has a comprehensive benefit package which covers over 95% of disease conditions in Ghana [38]. The benefit package however excludes treatment for cancers apart from breast and cervical cancers, HIV retroviral drugs, dialysis for chronic renal failure, hormone and organ replacement therapy and few others.

## Methods

### Study design and data

The study used data from a representative cross-sectional household survey in three districts. For financial reasons we selected only one district each to represent the three main ecological zones of Ghana (i.e. the southern, middle and northern zones). Three districts were Kwaebibirem in the southern zone, Asutifi in the middle zone and Savelugu-Nanton in the northern zone. In 2010, Kwaebibirem had a total population of 200,000 as against 114,029 in Asutifi and 139,283 in Savelugu-Nanton. The three districts were generally rural with agriculture as the main economic activity of the population. Kwaebibirem district is noted for industrial and small-scale mining of diamond and gold while Asutifi is noted for gold mining. While Kwaebibirem and Asutifi have similar socio-economic characteristics, Savelugu-Nanton is relatively poor. All the districts had well established DMHISs with over 70% of the population reported to have ever registered with the NHIS since its inception.

The key indicator of the survey was active membership in the NHIS in 2010 and this informed the determination of the sample size using the formula for sample size for estimation of a single proportion [39]. Assuming that 50% of households in each district were active members of the NHIS and with a confidence level of 95%, 5% margin of error and an estimated design effect of 2.0, a minimum sample size of 768 households per district was required for the survey. Since it was not possible to identify insured and uninsured households from the district schemes, it was decided to select a representative sample in each district in order to guarantee sufficient numbers of insured and uninsured households. In each district, EAs were stratified into rural and urban after which 27 representative EAs were sampled based on the Ghana Statistical Service’s classification and demarcations. All households in each EA were listed to obtain a sampling frame from which 30 households were randomly sampled. Selecting 30 households from each EA resulted in a total of 810 households per district (27 EAs × 30 households) which was closer to the estimated minimum required size of 768. In all, a total of 2,430 households were surveyed in 81 EAs in the three districts. Field interviewers had up to three visits per households if the household head was not available during the first visit. The response rate was 99.5% resulting in a total of 2, 418 households for the analysis. Data collection took place between February and April, 2011 by ten trained interviewers and two supervisors.

A structured household questionnaire was administered to the household head in a face-to-face interview by ten trained field assistants. The questionnaire had modules on demographic composition and socio-economic characteristics of the household, health status (e.g. illness in the last 6 months, presence of chronic illness), health insurance status of household members, reasons for non-enrolment and non-renewal, access to health services, household dwelling characteristics as well as assets ownership. In addition, data on annual household consumption expenditure consisting of expenditures on food and non-food items including health care and imputed values for home produced items such as food and housing as used in the Ghana Living Standard Surveys were collected [40]. The survey tool was developed specifically for this study. Field assistants were trained for seven days after which the survey tool was pre-tested. Based on the results of the pre-testing, final corrections were made to questionnaire before the main survey.

### Statistical analysis: analysis of the affordability of the NHIS contributions

The household was the unit of analysis for the study. A household is defined as a person or a group of persons, who live together in the same dwelling, sharing the same house-keeping arrangements and are catered for as one unit [40]. Households are not required to enrol as a single unit in the NHIS but the decision as to who gets to enrol in the household to a large extent is influenced by the household head who is a prominent decision maker regarding access to health and intra-household resource allocation [13,23,34]. Studies on household decision making have also observed that joint decision-making is quite common among married couples in Ghana on issues relating to healthcare, use of a partner’s earnings and major household purchases [41,42]. Three types of households were identified from the survey with respect to their health insurance status in the NHIS through the district schemes. A household is defined as uninsured if none of its members was insured with the NHIS, partially insured if at least one member was insured or fully insured if all the members were insured at the time of the survey.

The affordability analysis was based on the ‘normative definition’ of affordability which relates the total price of health insurance for all household members to the overall income of the household. Health insurance is defined as affordable to a household if purchasing it leaves enough income for it to meet its other socially defined minimum necessities required for living. This means that the total payment for health insurance plus the socially defined minimum level of spending on other socially required goods should be less than household’s total annual income [27,28,43]. Thus, using the ‘budget-based approach to affordability’ by Gruber and Seif [28], health insurance is affordable if ***a + b < c****,* where ***a*** is household’s minimum level of spending on necessities, ***b*** is the household total annual health insurance contributions and ***c*** is the household’s total annual consumption expenditure. In the absence of reliable household income data, we used total annual household consumption expenditure data from our survey as a proxy to household available resources [44]. The household total annual consumption expenditure was the sum of the monetary value of all items purchased by the household and home produced items meant for household consumption during the reference year for the survey as reported by the household.

The national upper poverty line of 370 Ghana cedis (GhȻ) (US$246.6 in March 2011) measured in 2006 [45] could not be used to represent the socially defined minimum level of spending on necessities because we considered it low and far below the World Bank’s poverty line of US$1.25 per day (US$456.25 per year). Instead, this study adopted the methodology by Xu [46] which uses a food-share based poverty line to estimate household subsistence spending. The method involves the estimation of household’s food expenditure share by dividing the household food expenditure by its total consumption expenditure. It also involves the estimation of equivalised food expenditures for each household to make it easy to compare welfare across households with difference size and demographic composition [47]. This adjusts for the fact that large households need larger food expenditures while taking into account that food consumption needs varies between adult and children and that the marginal cost of feeding one additional person is diminishing, i.e. economies of scale apply. To obtain the subsistence expenditure per adult equivalent, the total household consumption expenditure is adjusted for household size and composition using the formula: ***AE = (A + αK)***^***θ***^ where ***AE*** is the number of adult equivalents, ***A*** is the number of adults in the household and ***K*** is the number of children. The parameter ***α*** is the cost of a child relative to that of an adult while ***θ*** captures the effect of economies of scale [44,47]. Currently, there is no Ghana-specific adult equivalent scale, indicating parameter values. In a recent study on the progressivity of health care financing and incidence of service benefits in Ghana, Akazili et al. [48] used *α* = 0.5 for children between 0–14 years (≥15 years can be legally employed) and *θ* = 0.75 and this was adopted for our study. The food-share poverty line is then defined as the average food expenditures of households whose food expenditure share of total household consumption expenditure is within the 45th and 55th percentile of the total sample. By multiplying the subsistence expenditure per capita (i.e. the estimated poverty line) by the adjusted household size (*AE*), we estimated the total subsistence spending for each household. By these calculations, we derived a poverty line of GhȻ798.42 (US$532.17). A household was then identified as poor if the total household expenditure was smaller than the estimated poverty line. We also created socio-economic quintiles from the equivalised household consumption expenditure to represent the socio-economic status of the households.

Further, we computed the household total annual health insurance contributions (i.e. premiums and registration fees) required to be fully insured. The expected total annual health insurance contributions for each household depended on the total number of premium-paying adults in the household, the number of premium-exempt individuals who pay only the registration fee as well as the premium and registration fee for the district of residence. Data from the three districts showed that in 2010/2011, the registration fee per registrant were GhȻ3.0 in Asutifi, GhȻ4.0 in Kwaebibirem and GhȻ5.0 in Savelugu-Nanton. The premiums were GhȻ13.0 per person in Asutifi, GhȻ14.0 in Kwaebibirem and GhȻ12.0 in Savelugu-Nanton. This means the total contribution per a premium-paying adult in the informal sector was GhȻ16.0 in Asutifi, GhȻ17.0 in Savelugu-Nanton and GhȻ18.0 in Kwaebibirem. We multiplied the individuals in each household (i.e. premium-paying and premium-exempt individuals but registration fee paying) by their respective NHIS contributions as determined by the district schemes. The sum of these contributions for each household constituted its expected total annual NHIS contributions. The affordability analysis was then performed to identify all households which could not afford the expected NHIS contributions for all their members. These households were termed the ‘unafforders’. The unafforders were then categorised into ‘uninsured unafforders’ (i.e. uninsured households which would not be able to afford full insurance for their members) and ‘insured unafforders’ (i.e. insured households which were identified as unable to afford full insurance but have managed to have it) [27]. Significance tests were conducted to check for differences among uninsured, partially insured and fully insurance households. The choice of the statistical test depended on whether the variable was continuous (F-test of means because there were more than two groups) or categorical (Pearson’s chi-square test). All statistical analyses were performed in Stata 11.2.

### Ethical approval

Ethical approval for the survey was obtained from the Institutional Review Board (IRB) of the Noguchi Memorial Institute for Medical Research (NMIMR) of the University of Ghana with a certified protocol number of 069/11-12. Informed consent was also sought from all participating households.

## Results

### Descriptive statistics of surveyed households

A total of 11,089 household members were recorded in 2,418 households surveyed. About 28% of the households were categorised as fully insured and accounted for 23% of the household population. About 26% of the households were partially insured households and also accounted for 30% of the household members. The remaining 46% of the households were uninsured and had 47% of the household members. Table 1 presents descriptive statistics of the households comparing uninsured, partially insured and fully insured households. The results show that the households differed significantly in all the characteristics presented. The fully insured households were older (31.7 years), relatively had a higher number of household members with at least secondary education and had more formal sector workers though only 11% of the surveyed households had at least one formal sector worker. The partially insured households on the other hand had higher numbers of children, elderly and premium-paying adults. The uninsured households had fewer numbers of elderly, fewer formal sector workers and members with secondary or higher education. The results also show that the fully insured households reported higher illness per capita in the last six months prior to the survey and had a higher proportion (14.7%) of members with chronic illnesses. The uninsured reported the lowest illness per capita (0.28) and members with chronic illnesses (4.7%). A higher proportion of the fully insured households (66.2%) lived in urban areas compared to the partially insured (60.5%) and uninsured households (52.2%) (*p < 0.01*). The uninsured had a higher proportion (81.6%) of male heads than the partially insured (69.8%) and the fully insured (66.7%) households (*p < 0.01*). About 76% of all the household heads were married or had partners. About 22% of the heads of the fully insured households and about 20% of the partially insured households were divorced or widowed compared to only 14.7% among the uninsured. The heads of the uninsured households were the least educated while the fully insured relatively had better educated heads (*p < 0.01*). Most of the household heads (84.3%) were employed in the informal sector. Regarding socio-economic status (SES) of households, a higher proportion of the fully insured (52.51%) were in the top two socio-economic quintiles compared to only about 35% of the partially and uninsured households (*p < 0.01*). Significant proportions of the fully insured (72%) and partially insured (67%) households lived less than two kilometres from the nearest NHIS accredited health facilities compared to 55.5% of the uninsured. Nearly 22% of the uninsured were more than five kilometres from the nearest NHIS accredited health facilities. Finally, the Savelugu-Nanton district accounted for about 41% of the uninsured households compared to 32% in Kwaebibirem and 27% in Asutifi.

**Table 1:** **Descriptive statistics of households by their health insurance status**

**Characteristic**	**Household health insurance status**				**F-test/Pearson’s χ** ^**2a**^
	**Uninsured (n=1,117)**	**Partially insured (n=620)**	**Fully insured (n=681)**	**Total (n=2,418)**	
Household size & composition					
Mean age of household members (years)^b^	25.71 (11.5)	24.49 (10.5)	31.68 (18.2)	27.08 (14.2)	53.48***
No. of children under5 years (mean)	0.67 (0.8)	0.85 (0.9)	0.47 (0.7)	0.66 (0.8)	33.84***
No. of children between 5–17 years (mean)	1.66 (1.7)	1.83 (1.6)	1.32 (1.4)	1.61 (1.6)	17.88***
No. of ≥ 70 years in household (mean)	0.11 (0.3)	0.21 (0.5)	0.19 (0.4)	0.16 (0.4)	14.03***
No. of formal sector workers in household (mean)	0.09 (0.3)	0.12 (0.4)	0.18 (0.4)	0.12 (0.4)	11.21***
No. of premium paying adults in household (mean)	2.10 (1.3)	2.36 (1.3)	1.62 (1.1)	2.03 (1.3)	60.76***
No. with secondary educ. or higher (mean)	0.17 (0.5)	0.31 (0.7)	0.37 (0.7)	0.27 (0.6)	24.38***
Health status					
Household illness per capita (6mths)	0.28 (0.6)	0.46 (0.6)	0.64 (1.0)	0.43 (0.8)	48.25***
Household with a least one chronic illness (%)	4.74	10.81	14.68	9.20	53.48***
Household/Head characteristics					
Residence (%)					
Rural	47.81	39.52	33.70	41.73	35.94***
Urban	52.19	60.48	66.23	58.27	
Sex of head (%)					
Male	81.65	69.84	66.67	74.40	58.96***
Female	18.35	30.16	33.33	25.60	
Marital status of household head (%)					
Never married	8.77	1.61	6.02	6.16	48.55***
Married/in-union	76.54	78.23	72.25	75.77	
Divorced/widowed	14.68	20.16	21.73	18.07	
Education of household head (%)					
No formal education	45.03	42.90	32.01	40.82	65.14***
Primary	13.34	11.29	11.60	12.32	
Junior secondary/middle school	35.09	37.10	40.09	37.01	
Senior secondary or higher education	6.54	8.71	16.30	9.84	
Sector of employment of household head (%)					
Unemployed	4.57	7.58	10.43	6.99	54.71***
Formal	6.80	6.77	13.51	8.68	
Informal	88.63	85.65	76.06	84.33	
% of household heads belonging to social groups	28.65	35.65	39.65	33.54	24.62***
Socio-economic quintile (%)					
First	24.17	19.68	13.51	20.02	82.72***
Second	21.13	22.90	15.57	20.02	
Third	19.61	22.42	18.36	19.99	
Fourth	17.37	20.32	24.08	20.02	
Fifth	17.73	14.68	28.49	19.98	
Community/Health system factors					
Distance to nearest health facility (%)					
<2 kilometres	55.51	67.42	72.25	63.28	93.59***
Between 2–5 kilometres	22.65	21.29	21.00	21.84	
>5 kilometres	21.84	11.29	6.75	14.89	
District					
Savelugu-Nanton	40.82	32.74	22.17	33.50	75.40***
Asutifi	27.31	34.68	34.58	33.50	
Kwaebibirem	31.87	32.24	35.24	33.00	

Source: Household survey, 2011.

*Notes:* ^a^F-test for continuous variables (test of means) and Pearson’s chi-square (χ^2^) for categorical variables. Test of statistical significance: ***p<0.01.

^b^Standard deviations of continuous variables in parentheses (…).

### Reasons for non-enrolment in the NHIS

The results show that about 64% (634) of the uninsured and 47% (128) of the partially insured households reported that their members were not insured with the NHIS because they considered the contributions to be expensive. This was followed by 22.6% (223) of the uninsured and 37% (101) of the partially insured households who attributed their lack of insurance to the fact that their members did not fall sick to require a health insurance. About 14% (175) of the households also mentioned others reasons including perceived poor quality of care to the insured, registration difficulties, lack of trust in NHIS officials and inadequate benefit package, among others (Table 2). Among uninsured and partially insured households who found the NHIS contributions to be expensive, about 55% of them belonged to the two lowest socio-economic quintiles. About a quarter of the uninsured and partially insured households were also in the fourth and fifth socio-economic quintiles. For households who thought they did not need health insurance because their members do not fall sick, only about 10% and 14.6% of the uninsured and partially insured respectively were in the first socio-economic quintile.

**Table 2:** **Reasons for non-NHIS membership by socio-economic quintiles**

**Reason**	**Socio-economic quintile (%)** ^**a**^					**Pearson’s χ** ^**2b**^
	**First**	**Second**	**Third**	**Fourth**	**Fifth**	
**Premium/registration fee is expensive**						
Uninsured (n=634)	31.23	23.19	19.56	15.56	10.57	2.11
Partial insured (n=128)	28.13	27.34	19.53	17.19	7.81	
**Members do not fall sick**						
Uninsured (n=223)	10.31	20.18	24.22	17.49	27.80	7.45
Partial insured (n=101)	14.85	21.78	20.79	25.74	16.83	
**Other**						
Uninsured (n=131)	16.03	20.61	19.08	16.03	28.24	8.21*
Partial insured (n=44)	9.09	29.55	29.55	20.45	11.36	

Source: Household survey, 2011.

^a^Percentages are based on row totals.

^b^*Significant at 10%.

### Affordability of the NHIS premium and registration fee (i.e. NHIS contributions)

Table 3 presents summary statistics of the mean expected annual NHIS contributions if households were to become fully insured (full insurance). It also shows the estimated burden of the expected NHIS contributions on household expenditures. The mean expected annual NHIS contributions significantly differ among the districts (*p < 0.01*) and by the household’s health insurance status (*p < 0.01*). Savelugu-Nanton had expected NHIS contribution of GhȻ60.85 (US$40.56) compared to about GhȻ39.00 (UD$26.0) in the other two districts. This is because Savelugu-Nanton district had the highest registration fee (GhȻ5.0 compared to GhȻ3.0 in Asutifi and GhȻ4.0 in Kwaebibirem) and largest mean household size (5.7 compared to 4.1 in Asutifi and 3.5 in Kwaebibirem).

**Table 3:** **Summary statistics of the expected household NHIS contributions by selected variables**

**Variable**	**Mean** ^**a**^	**Std. Dev.**	**F-test** ^**b**^	**Household size (mean)**	**Number of premium exempt persons (mean)** ^**c**^	**Number of premium paying persons (mean)**	**Expected NHIS contributions as % of non-food expenditure (mean)**	**Expected NHIS contributions as % of total expenditure (mean)**	**Total (n)**
**District**			217.85***						
Asutifi	39.16	20.85		4.1	2.2	1.9	4.3	1.4	810
Kwaebibirem	39.44	22.66		3.5	1.9	1.6	4.6	1.5	798
Savelugu-Nanton	60.85	27.81		5.7	3.1	2.6	8.8	3.1	810
**Household insurance status**									
Uninsured	47.73	26.70	64.31***	4.5	2.4	2.1	6.7	2.2	1,117
Partially insured	53.69	25.22		5.3	2.9	2.4	6.2	2.0	620
Fully insured	38.01	23.16		3.6	2.0	1.6	4.2	1.5	681
**Socio-economic quintiles**			60.43***						
First	56.69	26.11		5.6	3.1	2.5	11.4	4.5	484
Second	51.26	27.05		5.1	2.9	2.2	7.0	2.1	484
Third	46.80	26.73		4.6	2.6	2.0	5.3	1.5	483
Fourth	44.67	25.35		4.2	2.3	2.0	3.7	1.1	484
Fifth	33.14	17.67		2.7	1.3	1.4	2.0	0.7	483
**Reason for non-membership**									
Payment is expensive			13.49***						
Yes	52.47	24.91		5.1	2.8	2.3	7.9	2.6	762
No	47.81	27.23		4.5	2.4	2.1	5.5	1.8	975
Does not fall sick			17.44***						
Yes	44.37	25.38		3.9	2.0	1.9	5.0	1.7	324
No	51.11	26.38		5.0	2.8	2.2	6.9	2.3	1,413
**All**	**46.52**	**26.02**		**4.4**	**2.4**	**2.0**	**5.9**	**2.0**	

Source: Household survey, 2011.

Notes:

^a^In Ghana cedis. US$1 = 1.5003 in March, 2011.

^b^Test of means. ***Significant at 1%.

^c^Exempted from paying the premium but they must pay the registration fee to be members of NHIS.

The results further show that the partially insured had the highest expected annual NHIS contribution of GhȻ53.69 (US$35.79), followed by the uninsured (GhȻ 47.73; US$31.81) with the fully insured households having the lowest (GhȻ38.01; US$25.33). As shown in Table 3, the partially insured household had the largest household size (5.3) while the fully insured households had the smallest household size (3.8). For all households, the expected annual NHIS contribution as a proportion of household non-food expenditure was 5.9% on the average and 2.0% of household total expenditure. The proportions were highest among the uninsured (6.7% and 2.2%) compared to the fully insured households (4.2% and 1.5%).

The expected annual NHIS contributions were significantly higher among households in the lower socio-economic quintiles (*p < 0.01*). For instance, households in the first socio-economic quintile had the highest expected annual NHIS contribution of GhȻ56.69 (US$37.79) compared to GhȻ33.14 (US$22.10) for households in the fifth quintile. For households in the first socio-economic quintile, the expected annual NHIS contribution was equivalent to 11.4% of the household non-food expenditure compared to just 2.0% for those in the fifth socio-economic quintile. The results further show that households which perceived the NHIS contributions to be expensive had a significantly higher expected annual contributions (GhȻ52.47; US$34.97) than those who did not complain about it (GhȻ47.81; US$31.87) (*p < 0.01*). Finally, households which thought their members were healthy and did not need health insurance had the lowest expected annual contributions which was significantly lower (GhȻ 44.37; US$29.57) than the other households (GhȻ51.11; US$34.07).

By the normative definition of affordability used for our analysis, 29% (702) of all the households surveyed were identified as unable to afford the expected annual NHIS contributions for full insurance (“unafforders”) (Table 4). Regarding their health insurance status, 34.5% (385) of the uninsured households were classified as uninsured unafforders while 30.2% (187) of the partially insured households were classified as unable to afford to be fully insured. Nearly 19% (130) of the fully insured households were also identified as insured unafforders.

**Table 4:** **Characteristics of unafforders by insurance status (%)**

**Variable**	**Household health insurance status**			**Total (n=702)**	**F-test/Pearson’s χ** ^**2**^
	**Uninsured (n=385)**	**Partially insured (n=187)**	**Fully insured (n=130)**		
**District**					
Kwaebibirem	16.62	17.65	12.31	16.10	3.59
Asutifi	23.64	28.34	26.92	25.50	
Savelugu-Nanton	59.74	54.01	60.77	58.40	
**Socio-economic quintiles**					
First	70.13	65.24	70.77	68.95	
Second	29.87	34.76	29.23	31.05	1.65
**Residence**					
Rural	52.47	42.25	37.69	47.01	10.84***
Urban	47.53	57.75	62.31	52.99	
**Household characteristics**					
Sex of household head					6.26*
Male	85.19	77.54	76.46	81.91	
Female	14.81	22.46	21.54	18.09	
Household size (mean)	5.5	5.9	4.9	5.5	6.24***
Expected contributions as % of non-food expenditure (mean)	10.7	10.1	8.6	10.2	6.30***
Expected contributions as % of total expenditure (mean)	4.0	3.6	3.6	3.8	3.46**

Source: Household survey, 2011.

Notes: *Significant at 10%; **Significant at 5%; ***Significant at 1%.

Table 4 shows the characteristics of the unafforders by their health insurance status. Savelugu-Nanton district accounted for 58.4% of the unafforders followed by Asutifi (25.5%) and Kwaebibirem (16.0%). This is not too surprising since the Savelugu-Nanton district is located in one of the poorest regions of Ghana. In terms of socio-economic status, all the unafforders were in the first and second socio-economic quintiles with almost 69% in the first quintile alone. Though more than half (53.0%) of the unafforders lived in urban communities, a higher proportion of the uninsured unafforders (52.5%) lived in rural communities. There was a higher proportion of male household heads (85.2%) among the uninsured unafforders compared to the other households. The results further show that the expected annual NHIS contribution of the uninsured unafforders was about 11% of their non-food expenditure compared to 10.1% for the partially insured and 8.6% for the insured unafforders. The unafforders also had a mean household size of 5.5, larger than the mean household size of 4.4 for all the households surveyed.

### Decision making on insurance allocation among partially insured households

The results have shown that the partially insured households had the largest household size, the highest numbers of children and elderly as well as the highest expected annual NHIS contributions. About 43% of them were in the first and second socio-economic quintiles. The study sought to find out individuals who were more likely to get insured in the NHIS in partially insured households. Females were more likely than males to insure (56.5% vs. 47.0%, *p < 0.01*) (Table 5). With the premium waiver for children and the elderly in place, a significant proportion of children and the elderly were insured (*p < 0.01*). Household members whose health status was perceived to be fair (59.6%) or poor (73.9%) were likely to be insured (*p < 0.01*). Though only 3.4% (113) of the household members had been diagnosed with chronic health conditions, about 83% of them were insured compared to 51% of those without chronic illnesses (*p < 0.01*). No significant difference was observed in the insurance status of informal and formal sector workers in partially insured households though formal sector workers were more likely to get insured.

**Table 5:** **Percentage distribution of insured and uninsured members of partially insured households by selected characteristics**

**Characteristic**	**Health insurance status**		**Pearson’s χ** ^**2**^	**Total** ^**a**^
	**Uninsured**	**Insured**		
**Sex**			29.76***	
Male	52.96	47.04		1,520
Female	43.48	56.52		1,810
**Age (years)**			50.33***	
Under 5	44.97	55.03		527
6–17	44.34	55.66		1,666
18–69	53.23	46.77		1,533
70+	25.95	74.05		131
**Perceived health status**			25.80***	
Excellent	50.82	49.18		1,216
Very good	48.81	51.19		1,467
Good	40.82	59.18		414
Fair	40.44	59.56		183
Poor	26.09	73.91		46
**Chronic illness**			45.03***	
No	48.90	51.10		3,217
Yes	16.81	83.19		113
**Sector of employment**			1.061	
Informal	47.94	52.06		3256
Formal	41.89	58.11		74

Source: Household survey, 2011.

Notes: ^a^Percentages are based on row totals.

***Significant at 1%.

## Discussion

Ghana’s NHIS is relatively young and faces many pressures to succeed [49]. Though enrolment has progressed over the years since 2004, the current active enrolment rate of 34% of the population is below expectation and a major challenge towards the attainment of universal coverage. There is every indication that the awareness level of the NHIS among households is high and the benefits of enrolment are well known [20-22,50]. The question that needs critical assessment is why enrolment is just about a third of the population even in the face of the comprehensive benefit package and generous exemption policy. This paper intended to examine whether affordability of the NHIS contribution at household level is a barrier to enrolment in the face of the persistent complaints about the NHIS contribution by households. This is an effort to understand the cost of having full insurance by households and the extent to which the NHIS contribution is a burden on household economic resources.

First of all, our results show that household size, the demographic composition of the household and its socio-economic status (SES) influence whether a household would become uninsured, partially insured or fully insured with the NHIS. While the size of the household and its demographic composition have direct effects on the total NHIS contribution, the SES of the household gives an indication of its ability to pay. Jehu-Appiah et al. [20] report of a negative effect of household size on enrolment in the NHIS. Our results show that the partially insured and uninsured households had larger household sizes and more children. Though uninsured and partially insured households could make good use of the existing premium exemption policy for children, the large numbers would still make the cost of full insurance higher. Policy to increase enrolment should aim at having special incentives to reduce the cost of full insurance for larger households.

The results of the affordability analysis suggest that about 66% of the uninsured and almost 70% of the partially insured households could afford to enrol all their members in the NHIS. Enrolling all household members in the NHIS is also not expected to exert a heavy burden on the majority of households as they would be expected to spend 5.9% of their non-food expenditures or only 2.0% of total expenditure on health insurance. This is close to the 5.0% and 1.2% reported by Diop [32] in the study in Senegal. A recent study [51] in Ghana suggests that about 30% of the 65% of the NHIS members who are exempted from paying premiums could indeed afford to contribute. It further adds that there were many people in the informal sector who have ‘simply not enrolled either because they do not understand the value of insurance or because they are healthy and unlikely to use services’ [51]. For such persons it is not about lack of ability to pay but possibly lack of willingness to pay because they may not see or have the need for it even if it is affordable as reported in this study by some “healthy” households. For these households, they would choose a particular insurance decision (e.g. uninsured, partially insured or fully insured) which maximises their expected utility weighing it against the cost of enrolment [52]. This observation is not peculiar in Ghana’s case. In the USA, Levy and DeLeire [26] report that even for persons in the same lowest expenditure group, the uninsured spent more on housing, alcohol, tobacco and education than the insured suggesting that they could possibly have paid for health insurance. This observation is also consistent with the finding by Bundorf and Pauly [27] which suggested that majority of the uninsured in the USA could afford to purchase health insurance.

Notwithstanding the finding that the majority (71%) of households can afford full insurance, the results also highlight the concern that enrolment in the NHIS is not pro-poor despite the premium exemption policy [19,20,24]. Households in the lower socio-economic quintiles were less likely to have full insurance and likely to complain that their NHIS contributions were expensive as shown by their significantly higher expected contributions. While the NHIS contribution would be affordable to households in the rich quintiles, the same cannot be said about households in the lower quintiles. For households in the first and second quintiles to use 11.4% and 7.0% of their respective total non-food expenditure on health insurance could impose a heavy burden on them. The fact that the households (29%) identified as unafforders were found in the two lower socio-economic quintiles calls for a review of the current flat rate of premium paid by all residents in a district irrespective of their socio-economic status.

It is known that people with low health status are more likely to demand health insurance [6,8,17]. This is because in voluntary insurance schemes where premiums are not related to individual risk levels, high-risk individuals see the premium as low given their high expected benefit from enrolment [27]. This may explain why partially insured households were more likely to insure their members with chronic health conditions or those whose health status were perceived to be fair or poor. The fully insured households also reported more chronic health conditions. These observations could be situated in Nyman’s argument that people purchase health insurance because of the access motive [53]. Thus, without health insurance, many households would not be able to gain access to expensive but most needed healthcare due to their inability to pay for healthcare. By this, it is possible that low income households which attach more value to good health would be more likely to buy health insurance because it helps them to ‘afford’ costly healthcare [54]. Perhaps this is why some households which were identified as unafforders had managed to have full insurance for all their members.

From the health insurance literature, the decision to buy health insurance in many LMICs is influenced by attributes of the insurer, health system factors and country specific contextual factors apart from the socio-economic status of households [4,6,12-18]. This is why in addition to addressing issues relating to affordability and equity in enrolment, it is equally important to give attention to the other reasons cited by about 14% of the uninsured and partially insured households for non-membership in the NHIS. The reasons such as perceived poor quality of health services, convenience of the enrolment procedure, lack of trust in scheme officials, inadequate benefit package, long distances to health facilities, negative provider attitude and other systemic factors associated with the NHIS need equal attention to attract more members [19,20,22,54-56]. A high proportion of the uninsured and partially insured households which made these complaints were in the fourth and fifth socio-economic quintiles. This means that many of such households could afford to enrol in the NHIS if the problems associated with the scheme are addressed adequately. In exploring how people’s perception about the NHIS influence enrolment and retention in the scheme, Jehu-Appiah et al. [20] observe that for the poor, favourable perceptions about the scheme relating to the benefits of the NHIS and convenience of scheme administration had significant and more positive effects on enrolment than perceptions about the price of enrolment. For instance, our results show that uninsured and partially insured households lived longer distances from NHIS accredited health facilities. As explained by Durairaj et al. [49] economically, rational people will not pay for services that are not available or are more difficult to access due to physical barriers to the extent they cannot derive the full benefits of membership.

### Limitations

This study has some limitations which could affect our results. First of all, the estimation of the expected annual NHIS contributions for households did not take account of indigents (i.e. destitutes) and pregnant women who are exempted from paying the premium and registration fee to enrol in the NHIS. Such persons were not captured by the household survey but having many of them in a household could reduce the cost of full insurance. Not all pregnant women in the households may however benefit from this exemption package either because they refuse to register for it or had enrolled prior to the pregnancy and therefore do not need a new one. We did not have such data.

It is also important to acknowledge that the normative definition of affordability used for the study is subjective and may have its own methodological and theoretical challenges as well as a lack of common acceptance of what constitute affordable premium [28,29]. For instance, some insured households categorised as “insured unafforders” could have borrowed to finance their enrolment which they may not be able to repay or could become a heavy burden on future household resources [28]. The consumption expenditure data used for the various measurements were self-reported by households and could suffer from recall errors which could bias the estimates, though quality control measures were adhered to during the data collection exercise. In addition to the use of the quantitative method, the issues of affordability could have been explored further by the use of qualitative methods. This would have made it possible to have a deeper understanding of the enrolment behavour of households. Finally, the lack of a more current national poverty line did not make it possible for us to assess how reliable our estimated poverty line is. Notwithstanding these limitations, the findings from the study are believed to reflect the general situation in the ecological zones the districts represented.

## Conclusion

This study has shown that majority of households without health insurance could afford full insurance with a minimum burden on their resources despite the complaints about the premium and the registration fee. Households which can afford to enrol all their members but have refused to do so must be enticed with innovative approaches to increase coverage. In the medium to long term, enforcement of the National Health Insurance Act which makes membership in the NHIS mandatory for residents of the country would be a positive step towards achieving universal coverage. One way of doing so could be to make the NHIS membership cards a requirement to access important services such as the acquisition of national passports, driving license and opening of bank accounts for adults as a way of getting more households to enrol in the NHIS. This would force people to register with NHIS, either by use of own means or by applying for exemptions. While relatively easy to implement in practice, such a policy may however meet political opposition and would need a well-developed exemption system to be in place.

Consideration should also be given to some of the factors which have direct effect on the cost of enrolment. Since large household size has negative effect on enrolment, abolishing of the registration fee for children who are exempted from paying the premium would be a positive step. For low resourced households who cannot afford full insurance, effective targeting of the premium exemption policy could benefit them. For instance, expansion of the livelihood empowerment against poverty (LEAP) program which is a cash transfer intervention to provide financial assistance to the extreme poor and vulnerable households which also gives them free enrolment in the NHIS could increase coverage of the scheme. Policy should also aim at reviewing the NHIS contributions to reflect the socio-economic status of households instead of the current flat rate for all which exert a heavy burden on poor households.

Finally, attention should be given to the non-financial factors which are equally inimical to enrolment in the NHIS. Addressing any institutional and systemic bottlenecks on the part of the insurer and service providers such as improvement in physical access to healthcare, reducing waiting time at health facilities and making the enrolment procedure more convenient to clients would enhance the drive towards achieving universal coverage of the scheme.

## Abbreviations

CHI: Community health insurance
DMHIS: District mutual health insurance schemes
IRB: Institutional Review Board
LMIC: Low-and middle-income countries
NHI: National Health Insurance
NHIA: National Health Insurance Authority
NHIF: National Health Insurance Fund
NHIS: National Health Insurance Scheme
NMIMR: Noguchi Memorial Institute for Medical Research
SSNIT: Social Security and National Insurance Trust
